# An experimental test on time constraint and sexual conflict over parental care

**DOI:** 10.1002/ece3.1620

**Published:** 2015-08-07

**Authors:** Matteo Griggio

**Affiliations:** Department of Biology, University of PadovaVia U. Bassi 58/B, I-35131, Padova, Italy

**Keywords:** Brood desertion, parental care, parental investment, *Petronia petronia*, sexual conflict

## Abstract

Because parental care is costly, a sexual conflict between parents over parental investment is expected to arise. Parental care behavior is an adaptive decision, involving trade-offs between remating, and consequently desertion of the brood, and continuing parental effort. If the main advantage of desertion is remating, then this will be a time constraint, because the deserting individual will require a certain minimum period of time to breed again in the same breeding season. So, a short breeding season should force certain individuals to desert the first brood to have enough time to successfully complete their second breeding attempt. The rock sparrow, *Petronia petronia*, is an unusual species in which brood desertion can occur in both sexes and the breeding season is quite short so it is a good species to investigate the role of time constraint on brood desertion. For 3 years, I investigated the brood desertion modality of the rock sparrow. Then, for 2 years, I removed a group of experimental nest boxes during the autumn. Later, I re-installed the experimental nest boxes after the start of the breeding season (2 weeks after the first egg was laid), mimicking a shortening of the breeding season for the (experimental) pairs that used experimental nest boxes. I found that in the experimental pairs, the percentage of deserting individuals was significantly higher than in the control groups, and the deserting individuals were older females. This experiment adds to our knowledge of timing of reproduction effects on individual decisions to desert by showing that a short and delayed breeding season may have different effects on males and females. To my knowledge, this is the first experimental study that demonstrates a direct link between time constraint and brood desertion.

## Introduction

The study of the evolution of parental care is one of the central focus in evolutionary ecology (Trivers 1972; Clutton-Brock 1991; Klug et al. 2013a,b). In particular, the amount of parental investment that the parent should provide to its young is a crucial decision (Clutton-Brock 1991; Houston et al. 2005). Because parental care is costly, a sexual conflict between parents over parental investment is expected to arise (Trivers 1972). In species where males and females cooperate to raise the young, one resolution of this conflict is to desert the offspring: One parent stops caring before the independence of the young. By deserting and remating in the same breeding season, a parent may increase its reproductive success, or the parent may improve its own survival reducing the cost, in time and energy, of caring for the brood deserted (Olsson 1997; Székely et al. 1999). On the other hand, helping the mate to raise the young may increase the fitness of its current offspring (Székely et al. 1996). Despite a long history of theoretical work on brood desertion (Clutton-Brock 1991; Székely et al. 1996; Webb et al. 1999; Houston et al. 2005) and despite the fact that brood desertion occurs in different animal classes (e.g., insects: Robertson and Roitberg 1998; fish: Jennions and Polakow 2001; birds: Valera et al. 1997; Roulin 2002; mammals: Kleiman 1977), few field experiments have manipulated variables that increase the probability of brood desertion. Some experimental studies manipulated the brood size (Beissinger 1990; Winkler 1991), the offspring quality (Erikstad et al. 1997), perceived paternity (Westneat and Sherman 1993), or attractiveness (Johnsen et al. 1997).

**Figure 1:**
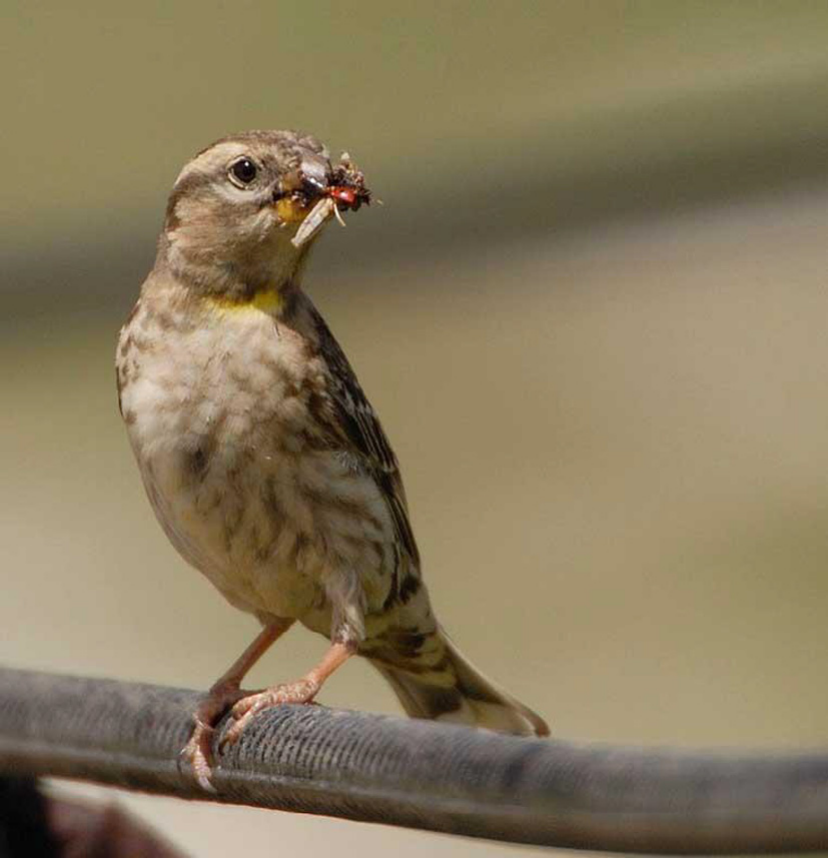
Rock sparrow, *Petronia petronia* (photograph by Adriano De Faveri).

If the main advantage of desertion is remating, then remating may be time constrained, because the deserting individual will require a certain minimum period of time to find a new mate and to breed a second time. So, a short breeding season should force certain individuals to desert the first brood to have enough time to successfully complete their second breeding attempt. Even though the time factor may be intuitively an important variable, to my knowledge, until now no experimental study was conducted to investigate the impact of time constraint on brood desertion. In the rock sparrow, *Petronia petronia*, (Fig.1) the breeding season is shorter than other multibrooded passerine species. For example, house sparrows (*Passer domesticus*) and tree sparrows (*Passer montanus*) in central–south Europe usually start to breed at the beginning of April till the end of July (Hoi et al. 2011; Poláček et al. 2013). The rock sparrow usually starts to breed at the end of May and ends at the end of July (Griggio et al. 2003; this study). In this species, brood desertion by both sexes occurs during the nestling feeding stage, and either parent is able to provide care on its own. The deserting partner usually remates successfully in the same breeding season (Pilastro et al. 2001; Griggio et al. 2005; Griggio and Pilastro 2007). Moreover, deserting females start to lay their first clutch about 1 week later, when the environmental conditions are better, than nondeserting females with two broods. Double-brooding females that deserted their first broods started their second broods about 1 week earlier compared with females that did not desert (Pilastro et al. 2001). So, even if the breeding period of rock sparrows is long enough for some individuals to produce two broods without the need of deserting the first brood, the breeding success is much lower than deserting individuals (Pilastro et al. 2001). Consequently, this species is a good subject to explore experimentally how a shortening in the breeding season might influence the brood desertion process. To shorten the breeding season of a group of pairs (experimental pairs), I postponed the start of the egg-laying period by installing a set of nest boxes later, when the rest of the population already started to lay eggs (control pairs). In this way, I experimentally created two groups of pairs in the same breeding season: the control pairs that started to breed as soon as they were ready and the experimental pairs that were forced to postpone their breeding phase because the breeding sites were available later. So, the experimental pairs experienced a delayed and shorter breeding season in comparison with control pairs.

This study explores for the first time experimentally the role of time constraint on parental care decisions in light of sexual conflict scenario. I predicted that a bigger percentage of experimental individuals than control individuals would desert their mates to have enough time to breed a second time in the same breeding season.

## Materials and Methods

### Study species

A wide array of mating patterns, including monogamy, polygyny, and sequential polyandry, have been recorded in this population as in other populations (Pilastro et al. 2001; Griggio et al. 2003; García-Navas et al. 2013). The rock sparrow shows a highly variable system of biparental care in which some males cooperate with females during the first week after hatching and then gradually decrease their food provisioning rate as the nestlings age. This can occur to the extent that, some days before nestlings are fledged, most of the provisioning is performed by the female (Griggio and Pilastro 2007). Approximately 20% of the males deserted the brood. More rarely, some males cared exclusively for the nestlings when their females deserted and started to lay a second clutch in another nest (ca. 10% of the females that successfully raised their first brood, see Results and Pilastro et al. 2001).

### Field work and experimental procedures

I studied a Sardinian population of rock sparrow, in Barbagia (40°23′30″N 9°12′12″E, 700 m a.s.l.). A mean of 36 nest boxes (range 32–40) were set up from 2008 (for more details see García-Navas et al. 2015). The population included approximately 20 breeding pairs each year (range 16–26). Nest boxes were also designed to work, when necessary, as traps so that adults could be individually color-ringed and measured. Each year I checked the nest boxes every second or third day from the start of the breeding season (May) until the last chicks fledged (July). I recorded pair bonds, laying date, and brood size. Since 2008, adults and fledglings have been marked with individual color ring combinations. Age of birds has been taken into account dividing individuals in two categories: first-year birds (young, ringed as fledglings the year before) and those at least 2 years of age (older). Individual body condition was calculated by dividing body mass by (tarsus length)^3^ owing to small values the body condition indices were multiplied by 10^4^ (Griggio and Hoi 2010). More details of the methods are given in Pilastro et al. (2001); Griggio et al. (2005); Griggio and Pilastro (2007).

In autumn (2010, 2011) prior to the experiments commenced, ten experimental nest boxes were removed each year. In spring (2011, 2012), the experimental nest boxes were reinstalled each year two weeks after the breeding season started (day in which the first egg was laid: end of May). Therefore, after the re-installation of the experimental nest boxes, the total number of nest boxes available remained the same (Table1). The total number of nest boxes was similar to the number of nest boxes during the pre-experimental years (2008–2010). Brood desertion was confirmed when one parent was no longer observed at the nest and was seen later in the field area or at a second nest (for more details, see Griggio and Pilastro 2007). Desertion was assumed to have taken place midway between the dates the partner was seen for the last time at the deserted nest and the subsequent observation (Griggio and Pilastro 2007).

**Table 1:** Number of nest boxes occupied and not occupied in the 2 years of experiment

	Control nest boxes (*N* = 29)		Experimental nest boxes (*N* = 10)	
Year	Occupied	Not occupied	Occupied	Not occupied
2011	28^1^	1	7	3
2012	29^2^	0	8	2

1 Fourteen pairs of rock sparrow and 14 pairs of Spanish sparrow.

2 Fifteen pairs of rock sparrow and 14 pairs of Spanish sparrow.

If not otherwise stated, differences were assessed using Student's *t*-test. Sample sizes vary across tests as incomplete data forced the exclusion of some individuals. Statistical analyses were carried out with SPSS 18 (IMB SPSS Statistics, NY). If not otherwise stated, means ± SD are given and all probabilities are two-tailed.

### Ethics statement

The experiments reported in this study comply with the current laws on animal experimentation in Italy and the European Union. The long-term nature of the study allowed me to confirm that handled birds and their offspring did not suffer any detectable reduction in welfare and survival.

## Results

### Desertion before the experiment

**Figure 2:**
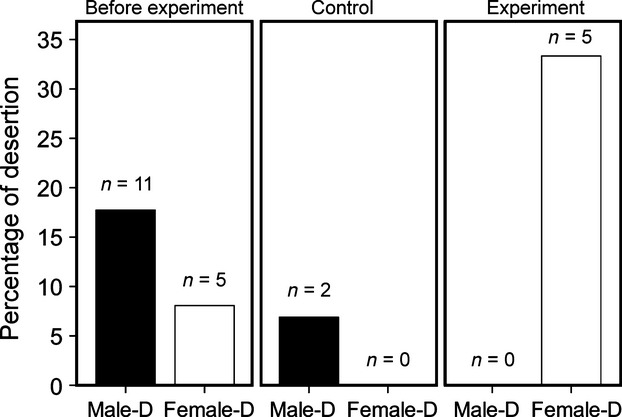
Percentage (%) of male (black bars) and female (white bars) brood desertion during the nonexperimental phase (years 2008, 2009, 2010) and during the experimental phase (2011, 2012). Sample sizes are given for each group.

Three years (2008, 2009, 2010) before the experiment commenced, I recorded the brood desertion rate in the study population. Out of the 62 breeding pairs followed, I observed five cases (one of unknown age, one young, and three older) of female brood desertion (8.06%) and 11 of male (three of unknown age, four young, and four older) brood desertion (17.74%; see Fig.2). Two out of the five deserting females remated with the same partner. In particular, these two deserting females started the new breeding event when their mates were still caring the nestlings from the first brood.

### Experimental desertion

In 2011 and 2012, almost all control nest boxes were occupied before the addition of experimental nest boxes (Table1). Approximately half of the control nest boxes were occupied by rock sparrows, the rest by Spanish sparrows (*Passer hispaniolensis*). Indeed, there is a strong competition for nest boxes in this study area (García-Navas et al. 2015). In 2011, seven experimental nest boxes were occupied by rock sparrows and eight in 2012. The occupation of the experimental nest boxes was immediate (few hours after installation, males started to sing on the roof of the nest box to attract a female, author pers. obs.). All the females that deserted started a second brood remating with another male. On the contrary, the two males that deserted were observed singing on a different nest, but they did not find a mate. In the 29 control pairs, two males (one young and one older) deserted (one each year, desertion rate: 6.9%). In the 15 experimental pairs, five females deserted (3 in 2011, 2 in 2012, desertion rate: 33.33%; see Fig.2). The difference between the two groups of pairs for the number of deserting individuals was statistically significant (Fisher's exact test *P *=* *0.036). In both years, the deserting females were all older females. Indeed, in 2011, the experimental nest boxes were occupied by three young females and four older females, and the three females that deserted were older females; in 2012 out of the four young and four older females that used the experimental nest boxes, the two females that deserted were older females (desertion rate for young females: 0%, desertion rate for older females: 62.5%, Fisher's exact test *P *=* *0.026). In both experimental years, there were more available males than in the three years before the experiment (the proportion of available mates at the time of desertion, period 2008–2010: 6.67, 11.76, 9.09; period 2011–2012: 30.56, 22.58).

Lastly, there was no significant difference in body condition between control and experimental individuals (males: t_42_ = 0.572, *P *=* *0.570; females: t_42_ = 0.121, *P *=* *0.913).

### Body condition and breeding success of all deserting females

The body condition of deserting females was better than nondeserting females (n_1_ = 10, n_2_ = 87; t_95_ = 2.06, *P *=* *0.042). There was no significant difference in body condition between young and older individuals (males: t_26_ = 0.930, *P *=* *0.361; females: t_52_ = 0.039, *P *=* *0.969). For deserting females, the interclutch time (days elapsed between start of laying of first and second clutch) was 35.5 ± 3.4 days, 9.7 days shorter than that for nondeserting double-brooded females (45.2 ± 3.3 days, t_20_ = 6.77, *P *<* *0.001). Finally, females deserting their first brood reared more young per year (8.2 ± 0.79, *n* = 10) than both single-brooded (4.2 ± 0.4, *n* = 60; t_68_ = 24.89, *P* < 0.001) and nondeserting double-brooded females (6.17 ± 1.34, *n* = 12; t_20_ = 4.57, *P *<* *0.001).

## Discussion

To my knowledge, this is the first study to show that an experimental manipulation of the time of breeding can influence the brood desertion decision and I found that the most important benefit of deserting females is remating. Indeed, the breeding success of deserting females was significantly greater than of both single-brooded females and double-brooded females that did not desert their first brood. Moreover, I found that older females deserted their offspring more often than younger females. So, two questions arise. Why in the experimental group, were only females, and not males, deserting? Why only older females deserted? There are two possible answers to the first question. First, males could be less sensitive to delayed breeding season because usually they are faster to desert their nests than females are (Pilastro et al. 2001). Second, it was already observed that in this species, the frequency of deserting females is positively correlated with the frequency of males available as mates at the time of desertion (Pilastro et al. 2001). In both experimental years in my population, I observed more available males than in the three years before the experiment: a particular favorable situation for females to desert. It can be argued that this explanation does not take into account the fact that the pattern was opposite in control pairs. One possible explanation is that the interactions between the number of potential mates available and the shortening of the breeding season (probably together with other factors like age and condition, see below) resulted in the decision to desert the current brood.

A possible answer to the second question is that the deserting females were also better in their physical conditions than the other females (Van Dijk et al. 2012). So, it is difficult to separate the condition effect from the age itself. Moreover, it is possible that the experience of females could play a role. Maybe more experienced females could risk more than younger females. Theoretically, it seems that my results are against the view that, as future breeding prospects decline with advancing age, old females are expected to invest more in current reproduction than young ones. Young females should be more inclined to desert than older females because the value of their current brood is lower and because their residual reproductive value is higher (Eldegard and Sonerud 2009). However, rock sparrow is a short-lived species (average life span of about 1.5 years, author pers. obs.), so individuals could be making decisions based more on current conditions, experience, and the actual breeding season, because the chances of breeding in the following year are in any case quite low. Interestingly, my results are in line with a recent long-term study of prothonotary warbler (*Protonotaria citrea*). This study has shown that older females are more likely to double brood in the same breeding season (Bulluck et al. 2013). It was suggested that younger females, and especially females in poor conditions, withhold breeding effort in the current breeding season trying to increase longevity and lifetime reproductive success (Forslund and Part 1995; Bulluck et al. 2013). It should be noted that, if females always benefit, in number of fledglings, from deserting as my results suggest, there should be a reason for why they do not desert when they reproduce early in the season. One possible explanation is that females can desert only if there are good chances that their mates stay and take care of the brood. For example, males that get their partner late in the season are generally more likely to care for the brood (as results from the experiment seem to indicate) affecting female propensity for desertion. It is probable, in fact, that the female's decision to desert or not is also affected by the behavior of her partner. Indeed, a previous study on this species has shown that males that will desert the brood guarded and courted their females significantly more than other males (Griggio and Venuto 2007). Previous studies together with these results indicate that pair member interactions are very important in the desertion process.

In conclusion, my study shows for the first time that a delayed breeding event increased the probability of brood desertion by older, and in better condition, females. On the contrary, the same effect was not observed for males, suggesting a strong difference between sexes for the parental care strategies adopted in the brood desertion context.
